# Supplementary material for: N-Terminal Sequences of Signal Peptides Assuming Critical Roles in Expression of Heterologous Proteins in Bacillus subtilis
Source: Microorganisms. 2024 Jun 23;12(7):1275. doi: 10.3390/microorganisms12071275 (PMC11278945; doi:10.3390/microorganisms12071275)
Supplement: Supplementary file 1 [file microorganisms-12-01275-s001.zip › microorganisms-3006447-supplementary.pdf]

N-Terminal Sequences of Signal Peptides Assuming Critical Roles in Expression of Heterologous Proteins in *Bacillus subtilis*

Meijuan Zhang [1,†], Jie Zhen [2,3,4,†], Jia Teng [2,5], Xingya Zhao [2,3], Xiaoping Fu [2,3], Hui Song [2,3,4], Yeni Zhang [5],
Hongchen Zheng [2,3,4,6,*] and Wenqin Bai [2,3,4,6,*]

[1] College of Life Science and Agriculture Forestry, Qiqihar University, Qiqihar 161006, China;
zhangmeijuan_002@163.com

[2] Industrial Enzymes National Engineering Research Center, Tianjin Institute of Industrial Biotechnology, Chinese Academy of Sciences, Tianjin 300308, China; zhen_j@tib.cas.cn (J.Z.)

[3] National Center of Technology Innovation for Synthetic Biology, Tianjin 300308, China

[4] Tianjin Key Laboratory for Industrial Biological Systems and Bioprocessing Engineering, Tianjin Institute of Industrial Biotechnology, Chinese Academy of Sciences, Tianjin 300308, China

[5] College of Food Science and Biotechnology, Tianjin Agricultural University, Tianjin 300392, China;
zhangyeni@tjau.edu.cn

[6] Key Laboratory of Engineering Biology for Low-Carbon Manufacturing, Tianjin Institute of Industrial
Biotechnology, Chinese Academy of Sciences, Tianjin 300308, China

* Correspondence: zheng_hc@tib.cas.cn (H.Z.); baiwq@tib.cas.cn (W.B.); Tel.: +86-022-84861933 (H.Z.)

† These authors contributed equally to this work and share first authorship.

Table S1. Primers used in this study.

Primers	Sequence (5'-3')
APL-F	CGCCATATGGAATTCATGGCGGGCAATGCAGATTAC
APL-R	CTAGCTAGCTTAATAGCTCGTCTTCAGCCAGTTGTC
LipA-F	CGCCATATGATGAAATTTGTGAAACGCAGAATTATTG
$LipA_{(YncM\ N5)}$-F	CGCCATATGATGGCTAAACCGCTGCGCAGAATTATTGCGCTGGTG
$LipA_{(YncM\ N7)}$-F	CGCCATATGATGGCTAAACCGCTGTCAAAAATTATTGCGCTGGTGACAATTCTG
LipA-R	CCGGAATTCCGCTTTCGCGCTCGGTTG
YncM-F	CGCCATATGATGGCTAAACCGCTGTCAAAAG
$YncM_{(LipA\ N5)}$-F	CGCCATATGATGAAATTTGTGAAATCAAAAGGCGGCATTCTGG
$YncM_{(LipA\ N7)}$-F	CGCCATATGATGAAATTTGTGAAACGCAGAGGCGGCATTCTGGTTAAAAAAG
YncM-R	CCGGAATTCCGCATCAGCTGCTGGCAG
WapA-F	CGCCATATGATGAAAAAACGCAAACGCAG
WapA-R	CCGGAATTCCGCCAGCACATCCGCCG
PelB-F	CGCCATATGATGAAATACCTGCTGCCGAC
PelB-R	CCGGAATTCGGCCATCGCCGGCTGGG
AmyX-F	CGCCATATGATGGTCAGCATCCGCCG
$AmyX_{(LipA\ N5)}$-F	CGCCATATGATGAAATTTGTGAAACGCAGCTTCGAAGCGTATG
$AmyX_{(LipA\ N7)}$-F	CGCCATATGATGAAATTTGTGAAACGCAGATTCGAAGCGTATGTCGATGAC
AmyX-R	CCGGAATTCCGGCGGTGTCATGATTTC
$APL_{(LipA\ N5)}$-F	CGCCATATGATGAAATTTGTGAAAGATTACAATCTGACCGGCTTC
pMA5-test-F	CTTTGCTGAGGTGGCAGAGGGC
pMA5-test-R	GCTTGCTTTCGAGGTGAATTAGCTTG
APL-qPCR-F	ATTCGGGATTACCAGCAACGGG
APL-qPCR-R	ATCCGTCTGATTGTTGCGCACC
danN-qPCR-F	GCACTTGCCGCAGATTGA
danN-qPCR-R	AATGCAAGACGGTGGCTATC
